# Probiotic and Antifungal Attributes of *Levilactobacillus brevis* MYSN105, Isolated From an Indian Traditional Fermented Food Pozha

**DOI:** 10.3389/fmicb.2021.696267

**Published:** 2021-07-05

**Authors:** Rakesh Somashekaraiah, Walid Mottawea, Adithi Gunduraj, Udit Joshi, Riadh Hammami, M. Y. Sreenivasa

**Affiliations:** ^1^Departmen of Studies in Microbiology, University of Mysore, Mysuru, India; ^2^Department of Microbiology and Immunology, Faculty of Pharmacy, Mansoura University, Mansoura, Egypt; ^3^School of Nutrition Sciences, Faculty of Health Sciences, University of Ottawa, Ottawa, ON, Canada

**Keywords:** probiotic, lactic acid bacteria, antifungal activity, fermented food, *Fusarium*

## Abstract

The use of probiotics and antifungal capabilities of the lactic acid bacteria (LAB) isolated from different niches is a strategy to prepare functional cultures and biopreservatives for food/feed industries. In the present study, LAB strains isolated from an Indian traditional fermented food, Pozha, were evaluated for their probiotic properties and biocontrol potential. A total of 20 LAB isolates were selected from Pozha samples collected aseptically and screened for their antagonistic activity against *Fusarium verticillioides*. Among the bioactive isolates, *Lacticaseibacillus brevis* MYSN105 showed the highest antifungal activity *in vitro*, causing some morphological alterations such as damaged mycelia and deformed conidia. Cell-free supernatant (CFS) from *L. brevis* MYSN105 at 16% concentration effectively reduced the mycelial biomass to 0.369 g compared to 1.938 g in control. Likewise, the conidial germination was inhibited to 20.12%, and the seed treatment using CFS induced a reduction of spore count to 4.1 × 10^6^ spores/ml compared to 1.1 × 10^9^ spores/ml for untreated seeds. The internal transcribed spacer (ITS) copy number of *F. verticillioides* decreased to 5.73 × 10^7^ and 9.026 × 10^7^ by *L. brevis* MYSN105 and CFS treatment, respectively, compared to 8.94 × 10^10^ in control. The *L. brevis* MYSN105 showed high tolerance to *in vitro* gastrointestinal conditions and exhibited high adhesive abilities to intestinal epithelial cell lines. The comparative genome analysis demonstrated specific secondary metabolite region coding for bacteriocin and T3PKS (type III polyketide synthase) possibly related to survival and antimicrobial activity in the gut environment. Our results suggest that *L. brevis* MYSN105 has promising probiotic features and could be potentially used for developing biological control formulations to minimize *F. verticillioides* contamination and improve food safety measures.

## Introduction

Contamination of food/feed products by mycotoxigenic fungi and the presence of mycotoxins have attracted the attention of the scientific and economic world due to the major economic losses and reduction in food/feed quality. The main sources of fungal colonization in food/feed originate from plants, primarily cereals used to formulate food/feed (Lee and Ryu, 2017; Nematollahi et al., 2019). It has been estimated by Food and Agriculture Organizations (FAO) that 25% of the world’s food/feed commodities are contaminated with mycotoxigenic fungi (Fraeyman et al., 2017).

The fungal species more often encountered with intoxication and mycotoxicoses in cereal-based food/feed belong primarily to three genera *Aspergillus*, *Fusarium*, and *Penicillium. Fusarium* species are the most common fungi associated with cereal contamination worldwide, affecting the quality and shelf-life of cereal-based products. The nutritional characteristics of cereal grains make them susceptible to contamination by *Fusarium* species (Deepa et al., 2016). For instance, *Fusarium verticillioides* is considered the main cereal pathogen, with a wide range of plant hosts and fumonisin production (Deepa and Sreenivasa, 2017). Fumonisins traditionally associated with cereals and cereal-based products can induce both acute and chronic toxic effects in poultry and animals. Fumonisin-contaminated cereal-based products have increased the incidence of esophageal and liver cancer (Khan et al., 2018; Arumugam et al., 2019) while causing neural discomforts in humans. In animals, fumonisin is associated with several mycotoxicoses, pulmonary edema in pigs, and decreased bodyweight, immune disorders, and liver sclerosis in poultry (Pósa et al., 2014; Vendruscolo et al., 2016). Fumonisin B1, in particular, is classified as carcinogenic to poultry and animals (Coppock and Dziwenka, 2015).

Safe elimination of this mycotoxigenic fungus from food/feed is of paramount importance because of their health-related issues and significant economic losses. Most physical and chemical strategies followed to reduce fungal contamination have been shown to be relatively ineffective or difficult to implement into the production process. Many fungicides used to control *F. verticillioides* contamination in cereal grains are conventional chemical agents, which negatively impact the environment and human health (Algharibeh and AlFararjeh, 2019). Therefore, biological control is the best alternative to these chemical agents, as they do not cause adverse effects on the environment while not being harmful to humans and livestock. Among the biocontrol agents, probiotic lactic acid bacteria (LAB) represent a potent and interesting strategy, as they are widely used in food/feed and are part of beneficial gut microbiota capable of producing antimicrobial metabolites. There are different reports of using bacteria to control mycotoxigenic *Fusarium* species (Nagaraja et al., 2016; Deepthi et al., 2017). However, probiotic LAB has not been used so far to control *F. verticillioides*.

Probiotics are live microorganisms used in food supplements, which confer beneficial effects on the host by colonizing the gastrointestinal tract (GIT) (Gibson et al., 2004). These microorganisms have important features such as antiallergic, anticancer, and antimicrobial properties; enhancing host immune function; or reducing lipid and serum cholesterol (Elbanna et al., 2018). For many years, fermented foods have been considered for their beneficial properties while being enriched with beneficial LAB (Behera et al., 2020), such as *Lactiplantibacillus plantarum*, *Lactobacillus acidophilus*, *Leuconostoc* species, and *Lacticaseibacillus casei* (Deepthi et al., 2016; Yadav et al., 2016; Zommiti et al., 2017; Muhammad et al., 2019).

South Indian tribes have an ancient tradition in food technology, with fermented food being very popular across the country and consumed at breakfast, lunch, and dinner. One such fermented food is Pozha prepared at the household level in the Kerala region, composed of coconut milk, rice, and coconut water, which is not well characterized and varies with the region of production. In order to improve the safety and quality of this food internationally, there is a need to explore their microbiota, particularly LAB as biopreservatives.

Many food matrices similar to Pozha are used to isolate LAB that are promising biocontrol agents due to their antagonistic activity against fungal species (Kharazian et al., 2017; Siedler et al., 2019) and their ability to detoxify mycotoxins (Chlebicz and Śliżewska, 2020). Their antifungal activity has been associated with the production of several bioactive metabolites, including organic acids, fatty acids, and peptides (Luz et al., 2017; Kwak et al., 2018). Likewise, different studies have reported the capacity of LAB to degrade mycotoxins to less toxic intermediate compounds and are used as biopreservatives in food/feed industries (Martinez Tuppia et al., 2017). In this context, the present study aimed to isolate new LAB strains from an Indian traditional fermented food Pozha and evaluate their probiotic features and *in vitro* antifungal activity against *F. verticillioides*.

## Materials and Methods

### Sampling and Isolation of Lactic Acid Bacteria From Pozha

Five different samples of Indian traditional fermented food Pozha were collected aseptically from different regions of Kerala state of India. A total of 10 g from each sample was suspended aseptically in 90 ml de Man Rogosa Sharpe (MRS) broth (pH 6.5) and homogenized by vortexing. Each sample was enriched by incubating anaerobically at 37°C for 48 h. Each suspension was then serially diluted in phosphate-bufferred saline (PBS), plated on MRS agar plates, and incubated for 48 h at 37°C under anaerobic conditions. Single isolated colonies were selected and purified by repeated streaking, then were grown overnight in MRS broth at 37°C and stored at −20°C in 25% (w/v) glycerol.

### Preliminary Screening and Characterization of LAB Isolates for Antifungal Activity

Antifungal activity of the LAB isolates against *F. verticillioides* MTCC 8792 was determined using the agar overlay method described by Magnusson and Schnürer (2001) with slight modifications. Briefly, the LAB isolates were inoculated in 3-cm lines on MRS agar plates and incubated at 37°C for 24 h in anaerobic jars. The plates were then overlaid with 10 ml of potato dextrose soft agar (PDA) containing 10^6^ spores/ml of *F. verticillioides.* These plates were incubated at 28°C for 4 days, and the clear zone of inhibition was observed. The LAB isolates showing significant antifungal activity were selected for further studies.

The bioactive LAB isolates were presumptively identified by phenotypic analysis like colony morphology, Gram staining, and catalase assay, as previously described (Somashekaraiah et al., 2019). The Gram-positive and catalase-negative isolates were tested for acid–bile tolerance, autoaggregation, and cell-surface hydrophobicity as per Yadav et al. (2016). Furthermore, the isolates were tested for their antimicrobial activity against indicator microorganisms. The most promising antifusarial isolate MYSN105 was selected for further phenotypic and genotypic characterization and presented in this study.

### Molecular Identification of LAB Isolate

The selected LAB isolate MYSN105 was characterized genotypically based on 16S ribosomal DNA (rDNA) sequencing (Belfiore et al., 2013). The genomic DNA isolated using Qiagen DNeasy blood and tissue kit (Qiagen, Hilden, Germany) was subjected to 16S rDNA amplification using the universal primers, Bact8F (AGAGTTTGATCCTGGCTCAG) (Edwards et al., 1989) and 1391R (GACGGGCGGTGTGTRCA) (Lane et al., 1985), and Sanger sequencing. The generated sequence was analyzed using the National Center for Biotechnology Information (NCBI) BLAST tool (Altschul et al., 1990). The sequences of *L. brevis* MYSN105 and other representative strains were aligned using the computer software MEGA X 10.1.8, while the phylogenetic tree was constructed using the neighbor-joining method (Kumar et al., 2018).

### Probiotic Characterization of *L. brevis* MYSN105

#### Antibiotic Susceptibility

The antibiotic susceptibility of the selected *L. brevis* MYSN105 was assessed according to the European Food Safety Authority (EFSA, 2012) technical guidelines. The overnight grown culture was inoculated onto the surface of MRS agar plates by swabbing, and the antibiotic MIC strips (Hi Media, Mumbai, India) were applied. Plates were incubated anaerobically at 37°C for 24 h. After incubation, the plates were observed for inhibition zones, and the antibiotic sensitivity was expressed as sensitive or resistant (Noda et al., 2019).

#### Antibacterial Activity

The inhibitory effects of *L. brevis* MYSN105 against bacterial pathogens *Escherichia coli* ATCC 25922, *Staphylococcus aureus* ATCC 6538, and Salmonella Paratyphi ATCC 9150 were measured using the agar-well diffusion method as per Ridwan et al. (2008).

#### Tolerance to Gastrointestinal Conditions

The viability of the *L. brevis* MYSN105 exposed to simulated GIT conditions was determined, according to Soares et al. (2019), with some modifications. The overnight culture of *L. brevis* MYSN105 in MRS broth was centrifuged at 8,000 rpm for 10 min. The cell pellets were washed twice with PBS and resuspended in the same buffer and serially diluted to obtain a final concentrate of 10^7^ CFU/ml. The cells were then exposed to the action of pepsin (3 g/L) and lipase (0.9 g/L), corrected to pH 2.0 using a 1 M solution of sterile hydrochloric acid (HCl) simulating gastric phase. To simulate enteric phase I, bile salts (10 g/L) and pancreatin (1 g/L) were added, and the pH was adjusted to 5 using a 1 M solution of sodium hydroxide (NaOH). Finally, to simulate enteric phase II, 10 g/L of bile salts and 1 g/L of pancreatin were added to the sample again, and the pH was adjusted to 7. The sample was maintained at 37°C on a shaker (200 rpm) for 3 h. Samples were collected at 0, 2, 4, and 6 h, subjected to serial dilution using PBS buffered at pH 6.5. Aliquots of 100 μl were plated onto the MRS agar and incubated anaerobically at 37°C for 24 h to determine the viable count. The experimental procedures were performed in triplicates. The survival rate of the *L. brevis* MYSN105 was expressed using the following formula:

$$\%\text{Survival}=\frac{N\log final\left(Time6h\right)}{N\log final\left(Time0\right)}\times 100$$

#### Adhesion Assay on Caco-2 and HT-29 Cells

Caco-2 (ATCC HTB-37) and HT-29 (ATCC HTB-38) cells were cultured in Dulbecco’s modified Eagle’s medium (DMEM) at 37°C under a 5% CO_2_ atmosphere. Cells were seeded in 24-well plates and used at their confluence (>90% estimated by microscopic observation, reached after 3 days) to avoid association with the well. Adhesion assay was adopted from Moroni et al. (2006). The overnight grown cell cultures were washed twice (6,000 × *g* for 5 min) and resuspended at 6 × 10^8^ CFU/ml in sterile PBS. Adhesion assay was carried out using 100 μl of *L. brevis* MYSN105 and 100 μl of DMEM without serum and added to each well to attain a 100:1 ratio of bacteria to cell lines. Plates were incubated for 2 h at 37°C under a 5% CO_2_ atmosphere, drained, and washed three times with sterile PBS. Cells with adherent bacteria were harvested using Trypsin–ethylenediaminetetraacetic acid (EDTA) for 10 min at 37°C, and 200 μl of DMEM media was then added. Adherent *L. brevis* MYSN105 was enumerated on MRS agar incubated anaerobically at 37°C for 24 h. The results were expressed as the adhesion index, which refers to the number of bacteria adhering to 100 Caco-2 and HT-29 cells.

#### Growth Kinetics of *L. brevis* MYSN105 and Acidification Profile

The growth kinetics and acidification profile of *L. brevis* MYSN105 were assessed as per Hammami et al. (2009) with modifications. Briefly, the strain was inoculated in MRS broth and incubated anaerobically at 37°C for 24 h, and absorbance at 600 nm and pH decrease were measured every 2 h using a spectrophotometer and pH meter. The values are represented as means of two independent experiments in triplicates.

#### Whole-Genome Sequencing of *L. brevis* MYSN105

DNA was extracted from an overnight culture of *L. brevis* MYSN105 in MRS broth using NucleoSpin Microbial DNA kit (Macherey-Nagel, Duren, Germany) as per the manufacturer. The extracted DNA was quantified with a Qubit fluorometer (Invitrogen, Carlsbad, CA, United States) and stored at −20°C till use. The whole-genome sequencing library was prepared using Nextera^*TM*^ DNA Flex Library Prep (Illumina; San Diego, CA, United States) as per its protocol. The prepared library was paired end sequenced (2 × 151 bp) in a 1/20 MiSeq run with Illumina MiSeq platform (NuGut Research Platform, University of Ottawa, Ottawa, ON, Canada) using 300 bp MiSeq Reagent Kit v2 (Illumina).

#### Genome Assembly and Analyses of *L. brevis* MYSN105

The generated reads were *de novo* assembled using the Velvet Assembler V1.0.0 incorporated in Illumina BaseSpace Sequence Hub (Illumina). Kraken (Wood and Salzberg, 2014) was used to assign the taxonomy to the generated reads. The assembled contigs were annotated using the Prokka V2.4.1 (Seemann, 2014) and Rapid Annotation using Subsystem Technology (RAST) server (Aziz et al., 2008; Overbeek et al., 2014; Brettin et al., 2015). Mining for secondary metabolite genes in the assembled genome was conducted via antiSMASH 5.0 (Blin et al., 2019). The assembled contigs were submitted to the Phage Search Tool Enhanced Release (PHASTER) (Arndt et al., 2016) to identify and annotate the prophage sequences *L. brevis* MYSN105 genome.

### Antifungal Activity of *L. brevis* MYSN105 and Its Cell-Free Supernatant

#### Agar-Overlay Assay

The antifungal activity of *L. brevis* MYSN105 was determined using the agar overlay method, as described above in preliminary screening.

#### Antifungal Activity by Microdilution Method

The antifungal assay was done using 96-well microtiter plates described by Hammami et al. (2009) with some modifications. The *L. brevis* MYSN105-CFS at different concentrations was diluted using PD broth and transferred to wells, which were subsequently seeded with 10^6^ spores/ml of *F. verticillioides* per well. The microplates were incubated at 28°C for 24 h, and the absorbance at 600 nm was measured every 20 min using The SPARK^®^ multimode microplate reader (Tecan, Männedorf, Switzerland). The antifungal activity was plotted in comparison to the positive control.

Minimum fungicidal concentration (MFC) values of *L. brevis* MYSN105-CFS was expressed as the percentage of the CFS used for dilution in PD broth corresponding to the lowest concentration that inhibited the growth of *F. verticillioides* (OD_600_ nm) after 24 h of incubation. The MFC values were reported as means of two independent experiments in triplicates.

#### Residual Bioactivity of MYSN105-CFS After Treatment With Heat, Proteinase K, or pH Neutralization

The residual bioactivity of MYSN105-CFS was assessed following treatments with protease, pH neutralization, and high temperature, as described by Boubezari et al. (2018). The CFS was tested for its susceptibility to proteinase K (1 mg/ml) treatment incubated at 37°C for 2 h, followed by enzyme inactivation at 80°C for 10 min. To assess the thermal stability, CFS was heated at 100°C for 15 min and then cooled to room temperature. The effect of pH on CFS was tested by adjusting the pH to 7 using sterile 1 M NaOH. After each treatment, the residual antifungal activity was determined in triplicates by the microdilution method, as described above. The untreated CFS was used as a control.

#### Fungal Biomass Inhibition Assay

The fungal biomass inhibition was carried out using *L. brevis* MYSN105 and its CFS, as described by Valan Arasu et al. (2013). To prepare CFS, *L. brevis* MYSN105 was inoculated in MRS broth and incubated at 37°C for 24 h, then fermented broth centrifuged (8,000 rpm for 10 min) and filter sterilized (0.22 μm syringe filter, Millipore, New York, NY, United States). One hundred-milliliter potato dextrose broth (PDB) flasks preinoculated with the strain at 10^6^ CFU/ml or CFS at different dilutions (4, 8, 12, and 16%) were inoculated with a 7-mm diameter fungal disk of *F. verticillioides*. The flasks were then incubated at 28°C for a week. The flask without strain or CFS was used as control. The fungal mycelial mat was harvested and dried in a hot air oven at 50°C for 2 h. The dry weight of the fungal biomass of each treatment was measured and compared to the control. The experiment was carried out in triplicates.

#### Conidia Germination Inhibition Assay

The 24-well microtiter plate was used to evaluate the inhibitory effects of *L*. *brevis* MYSN105 and its CFS on the conidial germination of *F. verticillioides* (Deepthi et al., 2016). To 1.0 ml 0.1 M PBS in the well, 100 μl of *L. brevis* MYSN105 (10^6^ CFU/ml) and 10^6^ spores/ml of *F. verticillioides* were added. Another reaction mixture of 16% CFS in 1.0 ml of 0.1 M PBS, along with 10^6^ spores/ml of *F. verticillioides*, was added into the well. The reaction mixture with 10^6^ spores/ml of *F. verticillioides* and 1.0 ml of 0.1 M PBS was considered a control. The microtiter plate was incubated at 28°C for 24 h. The conidial germination was observed periodically at 2, 4, 8, 16, and 24-h time intervals, and the germinated conidia were counted using a hemocytometer. The conidia germination percentage was calculated using the formula: (number of conidia germinated/total conidia counted) × 100.

#### *In vitro* qPCR-Based Antifungal Activity

The quantification of fungal growth was performed by quantitative PCR (qPCR). The *F. verticillioides* samples treated with *L. brevis* MYSN105 or its CFS were collected at different times for 14 days. The qPCR amplification was performed using 20 ng of DNA with 0.5 μM primers ITS1-F 5′-CTTGGTCATTTAGAGGAAGTAA-3′ (Gardes and Bruns, 1993) and ITS2-R 5′-GCTGCGTTCTTCATCGATGC-3′ (White et al., 1990) and 1 × SsoAdvanced Universal SYBR Green Supermix (Bio-Rad, Hercules, CA, United States) in a total volume of 25 μl. The reaction mixture contained 1x SYBR Green Mastermix, 0.5 μM of forward and reverse primer, 20 ng of template DNA, and ddH_2_O to the 25 μl reaction volume. Amplification conditions consisted of a preliminary step of 5 min at 95°C followed by 40 cycles at 95°C for 15 s and 60°C for 30 s. A no-template control for primer pair was included in the run. The results were analyzed using Bio-Rad CFX Maestro software and Microsoft Office Excel based on Cq values observed. The Cq values generated by qPCR were compared with the standard curve to quantify the internal transcribed spacer (ITS) copy number of *F. verticillioides* in different treatments.

#### *In situ* Biocontrol of *F. verticillioides* in Maize Seeds

Biocontrol assay of *F. verticillioides* in maize seeds was performed as per Yang and Chang (2010) with slight modifications. The maize seeds were surface sterilized using 2% sodium hypochlorite solution for 10 min and later soaked in sterile distilled water for 3 h. The maize seeds were further soaked in CFS for about 4 h at room temperature. The seeds (approximately 10 g) were then placed in sterile Petri dishes. The seeds were then spiked with 50 μl of *F. verticillioides* (10^6^ spores/ml), and the maize seeds without CFS treatment were considered as control. The plates were incubated at 28°C for 7 days, and the growth of *F. verticillioides* was observed.

### Statistical Analysis

The results are expressed as mean ± standard error (SE) of triplicate values of independent experiments. Analysis of statistical significance was performed with Student’s *t*-test using GraphPad Prism8, where appropriate *p* < 0.05 is considered significant.

## Results

### Isolation, Screening, and Molecular Characterization of Bioactive LAB Isolates

**FIGURE 1:**
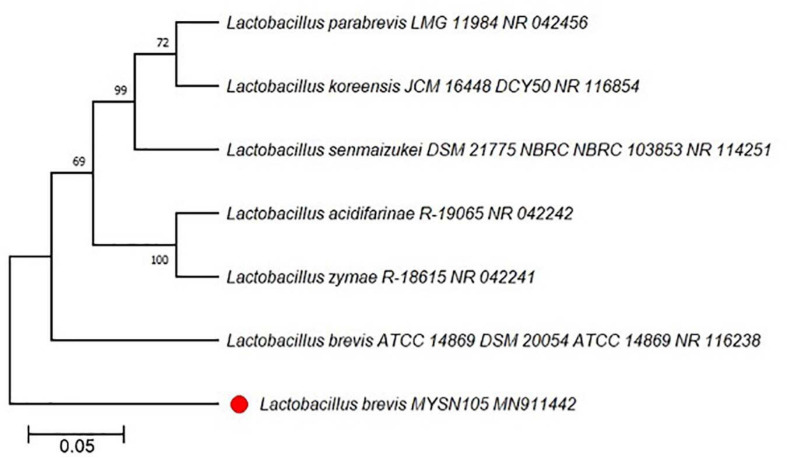
Phylogenetic tree showing the relative position of *Lactobacillus brevis* MYSN105 MN911442 marked with red taxon marker, using the neighbor-joining method of partial 16S rDNA sequences. Reference strains used for the comparison are labeled with their accession numbers of the 16SrDNA sequences. Bootstrap values for 100 replicates are shown at the nodes of the tree, using MEGA X 10.1.8. The scale bar corresponds to 0.05 U of the number of base substitutions per site.

After enrichment of microbes from Pozha, we obtained around 20 different isolates based on their colony morphology and catalase test. These isolates were subjected to screening for their antifungal activity (preliminary results), and the most potent isolate was selected for further characterization. The results confirmed that the MYSN105 isolate was a Gram-positive, rod-shaped, and catalase-negative bacterium. The MYSN105 isolate was non-hemolytic and was characterized as a hetero-fermentative LAB. The isolated MYSN105 was able to ferment glucose, xylose, arabinose, mannose, galactose, cellobiose, xylitol, arabitol, and lactose while producing organic acids (Table 1). The MYSN105 isolate was identified as *Lactobacillus brevis* (newly named as *Levilactobacillus brevis*) through Sanger sequencing of its 16S ribosomal RNA (rRNA) amplicon and sequence alignment (99.77% identity). The phylogenetic tree analysis was carried out to verify the isolate’s evolutionary position, which was constructed based on 16S rDNA sequences retrieved from the GenBank database (Figure 1). The 16S rRNA sequence of MYSN105 was submitted to GenBank under the accession number of *MN911442* and designated as *L. brevis* MYSN105 MN911442.

**TABLE 1:** Characterization of the isolate *MYSN105* from fermented food Pozha.

**Gram Staining**	**Gram-Positive Rods**		
**Catalase test**	**Negative**		
**Tolerance to acidic pH**	**Log CFU/ml^–1^**		
	**0 h**	**1 h**	**3 h**
pH 2	6.95 ± 1.31	4.15 ± 0.51	3.22 ± 1.20
pH 3	7.86 ± 1.52	6.36 ± 1.33	4.94 ± 1.01
pH 6	6.21 ± 2.34	6.13 ± 0.52	5.71 ± 1.91
**Bile salt tolerance**	**Log CFU/ml^–1^**		
	**0 h**	**4 h**	
2%	6.89 ± 0.52	5.29 ± 2.11	
4%	7.06 ± 1.14	6.17 ± 1.26	
**Hemolytic test**	**Negative**		
**Carbohydrate fermentation**	**Source**	**Growth**	
	Glucose	+++	
	Xylose	+++	
	Arabinose	++	
	Mannose	++	
	Galactose	+++	
	Cellobiose	++	
	Xylitol	+	
	Arabitol	+	
	Lactose	++	
**Cell surface hydrophobicity (%)**	65.90 ± 1.51		
**Auto aggregation (%)**	58.99 ± 1.34		
**Antimicrobial activity**	**Zone of inhibition (mm)**		
	***Escherichia coli* ATCC 25922**	***Staphylococcus aureus* ATCC 6538**	***Salmonella Paratyphi* ATCC 9150**
	18	21	19
**Survival in GIT conditions**	**Survival (%)**		
	**0 h**	**3 h**	**6 h**
	84.6 ± 0.61	68.4 ± 1.34	64.7 ± 2.01

*+ weak growth; ++ good growth; +++ very good growth. Data shown are mean ± SE of triplicate values of independent experiments.*

### Probiotic Features of *L. brevis* MYSN105

#### Resistance to GIT Conditions

As shown in Table 1, the *L. brevis* MYSN105 strain survived at pH 2, 3, and 6 for 3 h of incubation with cell count (log CFU/ml^–1^) of 3.22 ± 1.20, 4.94 ± 1.01, and 5.71 ± 1.91, respectively. The strain also exhibited a strong tolerance to bile salts for 4 h of incubation with cell count (log CFU/ml^–1^) of 5.29 ± 2.11 and 6.17 ± 1.26 at 2% and 4% bile salt concentration, respectively. Likewise, in the presence of GIT digestive enzymes, the survival rate of the strain after 6 h of incubation was 64.7% (Table 1).

#### Antibiotic Susceptibility of *L. brevis* MYSN105

The antibiotic susceptibility of *L. brevis* MYSN105 was tested against various antibiotics generally recommended by EFSA guidelines. The results were obtained by measuring the inhibition zone, indicating the minimum inhibitory concentration (MIC) at the intersection point between the inhibition zone and the strip edge. Compared to the MIC breakpoints recommended by EFSA, the MYSN105 strain was sensitive to ampicillin, gentamicin, erythromycin, clindamycin, tetracycline, and chloramphenicol while being resistant to kanamycin and streptomycin (Table 2).

**TABLE 2:** Susceptibility of *L. brevis MYSN105* to antibiotics.

**Antibiotic**	**MIC (μg/ml)**		Antibiotic susceptibility*
	***L. brevis MYSN105***	**MIC breakpoint recommended by EFSA**	
Ampicillin	0.75 ± 0.08	2	S
Gentamicin	4 ± 0.23	16	S
Kanamycin	–	64	R
Streptomycin	–	64	R
Erythromycin	1 ± 0.12	1	S
Clindamycin	2 ± 0.45	4	S
Tetracycline	8 ± 0.47	8	S
Chloramphenicol	2 ± 0.40	4	S

*(S) and (R): susceptible and resistant, as per EFSA guidelines. Data shown are mean ± SE of triplicate values of independent experiments.

#### Adhesion to Intestinal Cell Lines

The adhesion of *L. brevis* MYSN105 to Caco-2 and HT-29 cells was evaluated along with autoaggregation and cell surface hydrophobicity. The adhesion index of *L. brevis* MYSN105 to Caco-2 cells was estimated as 659.66 and to HT-29 cells as 1,066. Besides, the autoaggregation capability of *L. brevis* MYSN105 was 58.99%, while the cell surface hydrophobicity of MYSN105 was 65.90%.

#### Growth Kinetics and Acidification Profile

**FIGURE 2:**
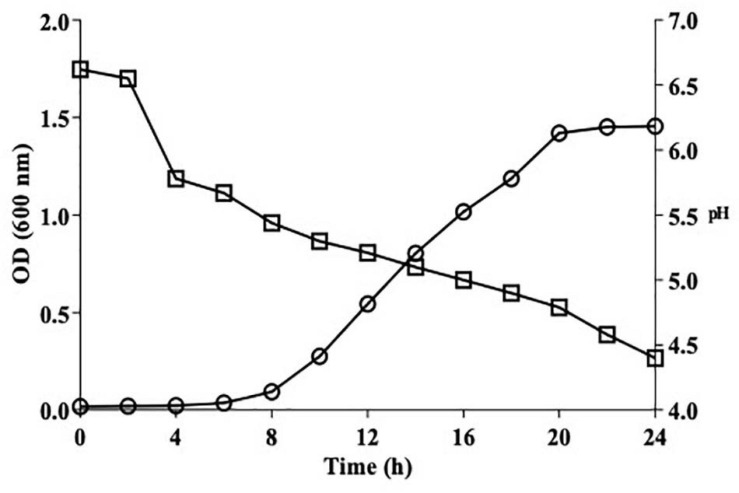
Growth kinetics (circle) and acidification profile (square) of *L. brevis* MYSN105 in MRS broth at 37°C for 24 h.

Figure 2 illustrates the growth kinetics of *L. brevis* MYSN105 with the pH variation for 24 h. The stationary phase was attained after 20 h of incubation, reaching the maximum growth, and OD remained stable up to 24 h. The acidification profile was assessed using pH measurement, which decreased from 6.7 to 4.79 as growth reached the stationary phase and 4.4 at 24 h.

#### Genome Characteristics of *L. brevis* MYSN105

**FIGURE 3:**
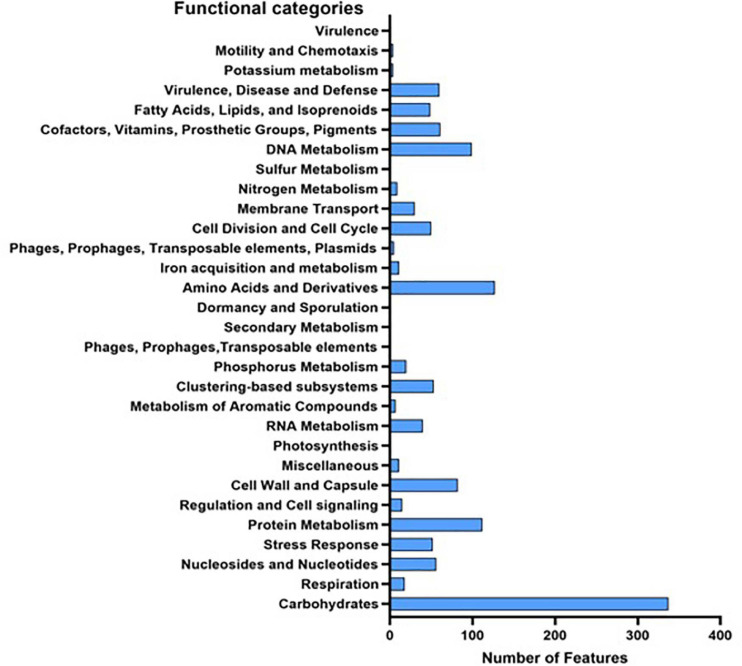
Functional categories of all predicted genes in *L. brevis* MYSN105 genome based on RAST and Prokka annotation.

While Kraken assigned 95% of the reads to *Lactobacillus*, 84% of the generated reads were assigned to *L. brevis*. Those reads were assembled in a draft genome of size 2,097,265 base with 46.27% GC content. Prokka annotation predicted 1,849 genes, 1,834 of which are protein-coding genes. Of the genes, 1,096 have a non-hypothetical function, and 491 with seed subsystem ontology. On the other hand, RAST annotation identified 2,926 CDs, with 1,315 assigned a functional category (Figure 3). AntiSMASH database identified two secondary metabolite regions, one for bacteriocin and the second for T3PKS (type III polyketide synthase). Four incomplete prophage sequence regions (3.8–7.6 kb) were identified in the genome, all belong to the PHAGE_Lactob_LBR48_NC_027990. The assembled genome was deposited in GenBank under bioproject accession number PRJNA679667.

### Antimicrobial Activity of *L. brevis* MYSN105 and Its CFS

#### Antibacterial Activity

The antibacterial effect of *L. brevis* MYSN105 was also tested on different foodborne bacteria, and the effective inhibition is summarized in Table 1.

#### Antifungal Activity on *F. verticillioides*

**FIGURE 4:**
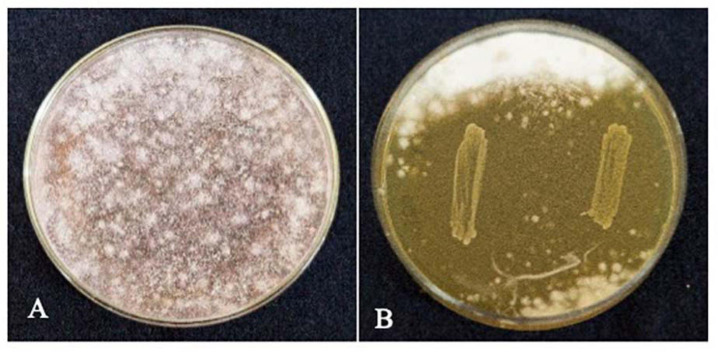
Inhibition of *F. verticillioides* by *L. brevis* MYSN105 by agar overlay method after 4 days incubation at 26°C. **(A)** Control *F. verticillioides*. **(B)** *L. brevis MYSN105* overlaid by *F. verticillioides*.

**FIGURE 5:**
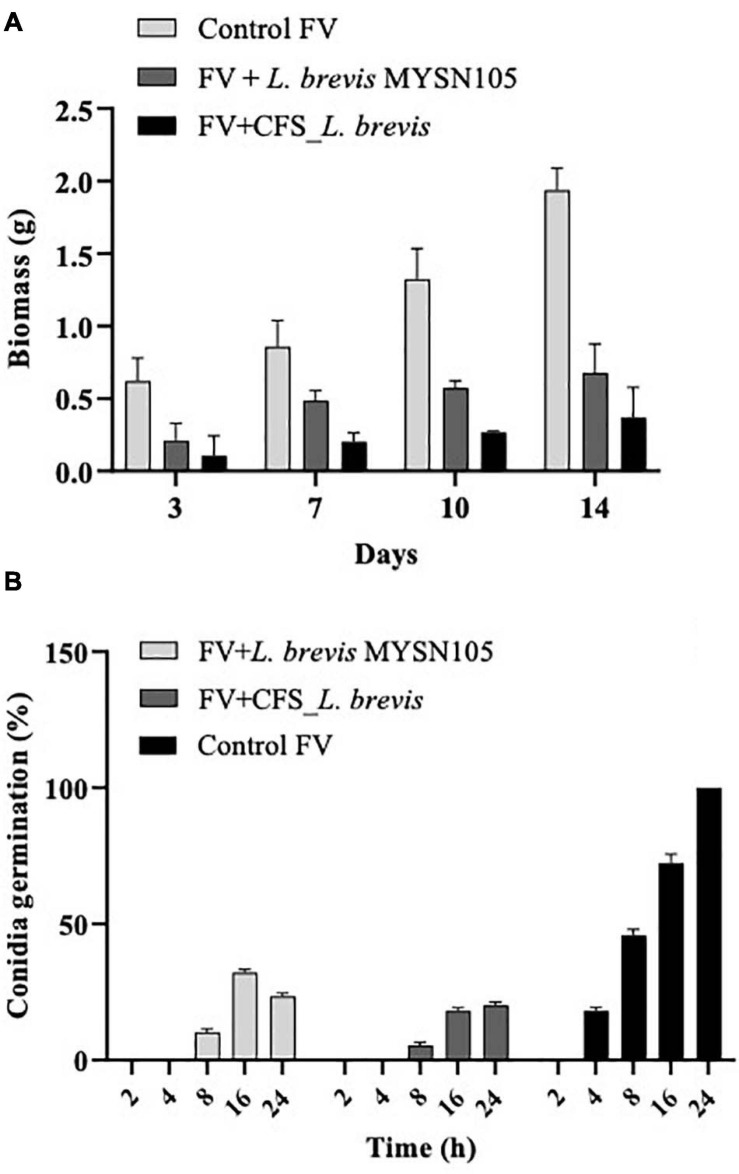
Effect of *L. brevis* MYSN105 and its CFS on fungal biomass and conidia germination. **(A)** Fungal biomass reduction by *L. brevis* MYSN105. **(B)** Conidia germination inhibition by *L. brevis* MYSN105. Data shown are mean ± SE of triplicate values of three independent experiments.

**FIGURE 6:**
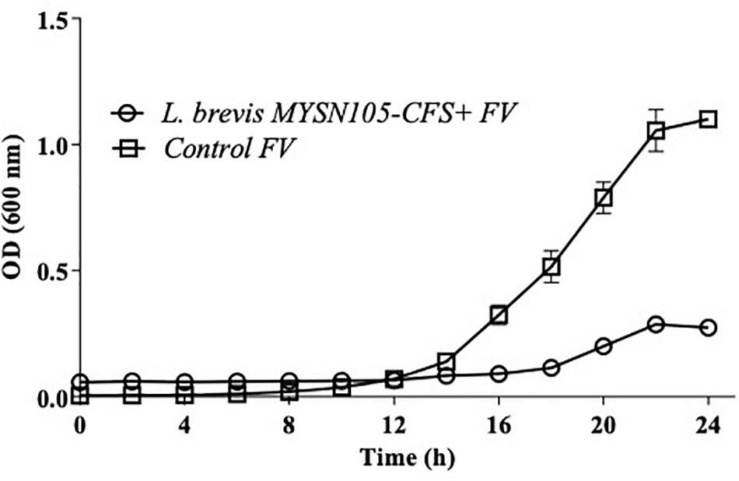
Antifungal activity of *L. brevis* MYSN105 against *F. verticillioides* in a microtiter plate for 24 h. Data shown are mean ± SE of triplicate values of three independent experiments.

**FIGURE 7:**
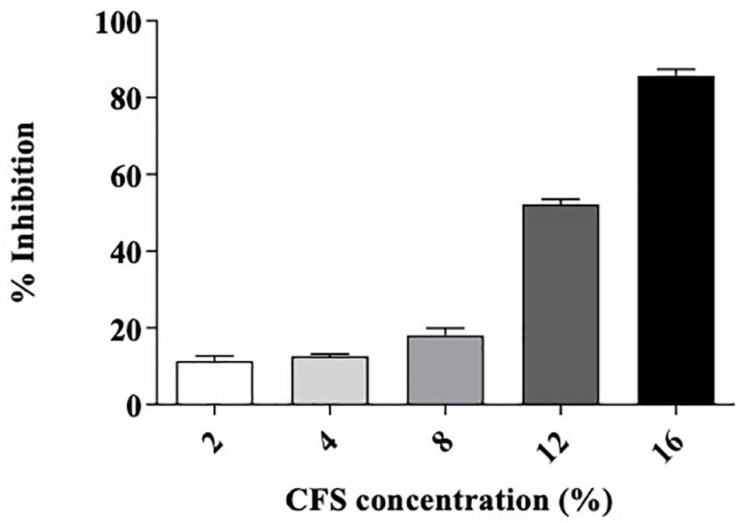
Minimum fungicidal concentration of *L. brevis* MYSN105-CFS against *F. verticillioides* by microdilution method. Data shown are mean ± SE of triplicate values of three independent experiments.

**FIGURE 8:**
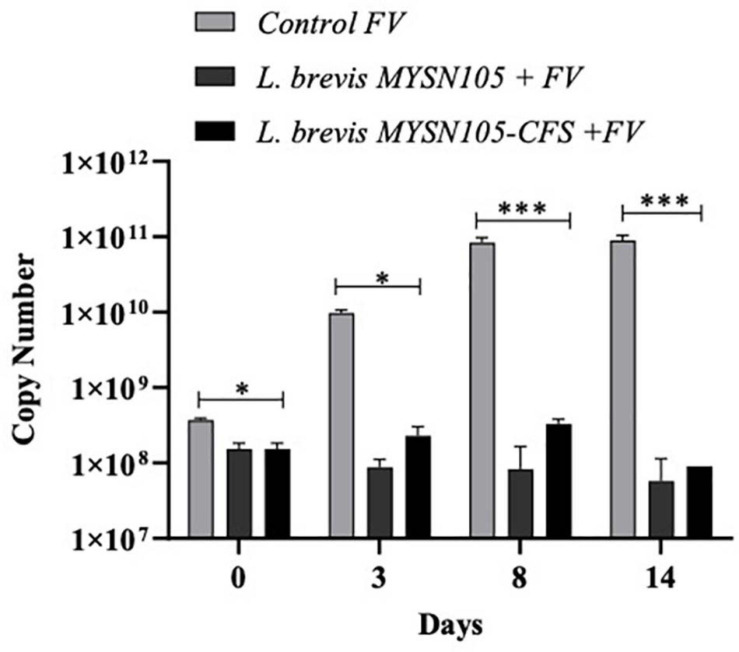
Quantitative PCR amplification of *F. verticillioides* using ITS primer. ITS copy number quantified using Cq values. Data shown are mean ± SE of triplicate values of two independent experiments and differ significantly (^∗^*p* < 0.05, ^∗∗∗^*p* < 0.001).

The strain *L. brevis* MYSN105 exhibited potent inhibitory activity against *F. verticillioides*, as shown in Figure 4. The strain also reduced the mycelial biomass of the target fungus, with biomass reduced to 0.679 ± 0.20 g after 14 days of incubation compared to the control (1.938 ± 0.15 g) (Figure 5A). In addition, the CFS from *L. brevis* MYSN105 strongly inhibited the *F. verticillioides* spore formation (Figure 6). The CFS showed a dose-dependent inhibition of *F. verticillioides* (Figure 7). The MFC was found to be 16% of CFS, inhibiting 85.71% of the spores compared to control. Likewise, after 14 days of treatment with 16% CFS, the biomass of *F. verticillioides* was highly decreased (0.369 ± 0.21 g) compared to control (1.938 ± 0.15 g) (Figure 5A). The absolute quantification of fungal growth in the presence of *L. brevis* MYSN105 and its CFS was also performed by qPCR. The results indicated that the fungal ITS copy number was decreased to 5.73 × 10^7^ and 9.026 × 10^7^ in the presence of *L. brevis* MYSN105 or CFS, respectively, in comparison to 8.94 × 10^10^ copies of the positive control (Figure 8).

#### Conidia Germination Inhibition Assay

The *L. brevis* MYSN105 and its CFS caused irreversible damage to conidia, resulting in cell mortality and hyphal growth inhibition (Figure 5B). After 24 h incubation, conidia showed deformation and were reduced to 23.41% ± 1.12 and 20.12% ± 1.15 in the presence of the strain or its CFS, respectively.

#### Sensitivity of the CFS From *L. brevis* MYSN105 to Proteinase K, pH Neutralization, and Heat Treatment

**FIGURE 9:**
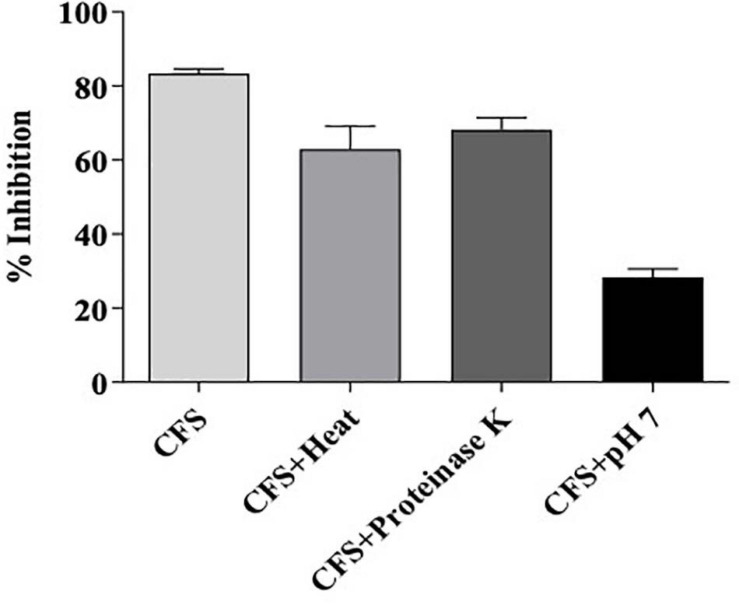
Residual bioactivity of *L. brevis* MYSN105-CFS against *F. verticillioides* after treatment with heat, proteinase K, or pH neutralization. Data shown are mean ± SE of triplicate values of three independent experiments.

The effect of proteinase K, pH neutralization, and heat treatment on the antifungal activity of *L. brevis* MYSN105-CFS is presented in Figure 9. The antimicrobial activity was moderately sensitive to proteinase K. The antifungal activity of CFS decreased at neutral pH 7, while a partial inactivation of the CFS was observed in heat treatment at 80°C for 20 min.

#### Biocontrol of *F. verticillioides* in Maize Seeds

**FIGURE 10:**
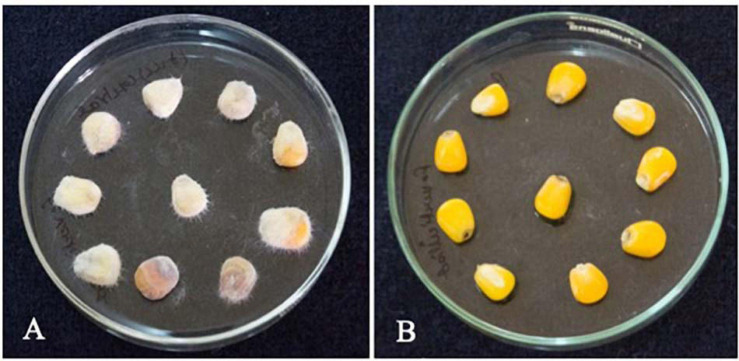
Effect of *L. brevis* MYSN105-CFS treatment on maize kernels inoculated with *F. verticillioides* after 7 days. **(A)** Control, maize kernel infected with *F. verticillioides*; **(B)** maize kernel treated with *L. brevis* MYSN105 CFS and infected with *F. verticillioides*.

The treatment of maize seeds using the CFS of *L. brevis* MYSN105 highly inhibited the *F. verticillioides* growth, with no mycelium formation after 7 days of ambient incubation conditions compared to control (Figure 10). The spore count in the control maize samples treated with fresh MRS broth reached 4.1 × 10^2^ spores/ml.

## Discussion

The current study investigated an unexplored niche Pozha for the presence of potent LAB having antifungal properties and desirable probiotic features. The Pozha samples were prepared using coconut milk, rice, and coconut water, fermented naturally, and later collected aseptically to isolate bioactive LAB. Among 20 isolates from different Pozha samples, a few were found to have antifungal activity against *F. verticillioides.* Of particular interest, *L. brevis* MYSN105 was highly potent against *F. verticillioides* while exhibiting attractive probiotic attributes. Taroub et al. (2019) and Judkins et al. (2020) showed that LABs are found in diverse ecological niches, including fermented food products, where they play an important role in nutrition and maintaining gut health. They are also promising biological control agents reported in several studies for their antifungal activity while being generally regarded as safe (GRAS). Our results are concomitant to other studies (Laitila et al., 2002; Oliveira et al., 2015; Deepthi et al., 2016), which demonstrated the efficiency of different LAB strains against some *Fusarium* species such as *F. avenaceum*, *F. proliferatum*, *F. graminearum*, and *F. culmorum*.

The candidate *L. brevis* MYSN105 exhibited interesting probiotic characteristics, including an excellent survival in acid and bile conditions in accordance with previous studies by Zommiti et al. (2017) and Soares et al. (2019). Besides, *L. brevis* MYSN105 was able to utilize a wide range of carbon sources, namely, glucose, xylose, arabinose, mannose, galactose, cellobiose, xylitol, arabitol, and lactose, with acid production, desirable features in food industries as probiotics (Ramesh et al., 2015; Lee et al., 2017; Kavitha et al., 2018). Probiotic features also include the ability to colonize and adhere to epithelial cells and mucosal surfaces (Lee and Salminen, 1995; Jacobsen et al., 1999); this helps them resist fluctuation of their intestinal level and inhibit the attachment of pathogenic bacteria via competitive adhesion all over the intestine nullifying inflammatory reactions (Guo et al., 2010; Sim et al., 2015). Therefore, autoaggregation and hydrophobicity determine the adhesion ability of probiotic bacteria (Collado et al., 2008; Meidong et al., 2017). In this study, *L. brevis* MYSN105 showed better hydrophobicity and strong autoaggregation, suggesting higher adhesion to epithelial cells. The adhesion results on Caco-2 and HT-29 cells demonstrated the potent adhesion capacity of *L. brevis* MYSN105 to the gastrointestinal tract. Previous reports have shown that adhesion of *Bifidobacterium* and *Lactobacillus* strains to intestinal mucus plays an important role in Enteropathogen exclusion (Collabo et al., 2005; Jung et al., 2019). Therefore, the antimicrobial activity exhibited by *L. brevis* MYSN105 provides a competitive inhibition of pathogenic microbes in the colonic environment. In addition, our probiotic candidate was non-hemolytic and sensitive to ampicillin, gentamicin, erythromycin, clindamycin, tetracycline, and chloramphenicol as per EFSA guidelines (EFSA, 2012). Hemolytic activity and antibiotic susceptibility are safety prerequisites for selecting probiotic strains (Argyri et al., 2013). Hemolysin is considered a virulence factor that can initiate infection and antibiotic resistance in bacterial strains having serious consequences on the host (Ramesh et al., 2015; Nandi et al., 2017). The genomic analysis is an important tool to help understand the functional properties of probiotic strains, including their safety, thus validating their efficacy (Qureshi et al., 2020). *L. brevis* MYSN105 was comprehensively studied for potential probiotic traits using whole-genome sequence analysis, which enabled identifying genes linked to safety and antimicrobial properties. Several studies have reported *Lactobacillus* species consisting of genes related to their stress resistance, antimicrobial properties, and other functional mechanisms (Chen et al., 2019; Toropov et al., 2020). The genomic finding suggests that understanding the functional and genetic characteristics could provide new insights into applications in gut health and animal feed additives (Heo et al., 2018). These attributes make *L. brevis* MYSN105 an attractive probiotic candidate (Jung et al., 2019) for industrial applications.

Food industries apply physical and chemical treatments to control mycotoxigenic fungi in food and feed (Faucet-Marquis et al., 2014). However, these treatments could reduce the nutritional value, safety, and sensory quality of food. Moreover, these methods require expensive equipment for the industry (Pierides et al., 2000; Kim et al., 2017). Many researchers have studied the management of mycotoxigenic fungi in food and feed using biological treatments such as probiotics microorganisms by assimilation or degradation approaches (Kim et al., 2017). Indeed, the biological approaches are increasingly explored due to their higher effectiveness and safety for humans (Jaibangyang et al., 2020; Rodríguez and Núñez, 2020). Our probiotic candidate *L. brevis* MYSN105 and its CFS both reduced significantly the growth of mycotoxigenic fungi, with findings from this study are in good accordance with previous investigations (Nagaraja et al., 2016; Deepthi et al., 2017; Plocková et al., 2018).

The neutralization of CFS from *L. brevis* MYSN105 reduced the antifungal activity of *F. verticillioides*, indicating a potential role for organic acids in the exhibited inhibition (Mauro and Garcia, 2019). Previously, the synergistic effects of different mixtures of identified compounds in *L. plantarum*, *L. sanfrancisco*, and *Weissella cibaria* were studied by Corsetti et al. (1998), Niku-Paavola et al. (1999), and Ndagano et al. (2011). The studies reveal that the combined effects of the compounds is much higher than the individual activities of the compounds (Lynch et al., 2014). The significant activity of *L. brevis* MYSN105 against *F. verticillioides* could be attributed to the combined effects of the organic acids. The CFS from *L. brevis* MYSN105 reduced the fungal growth and caused morphological alterations by inhibiting conidia, indicating that the antifungal activity is related to the bioactive compounds present in the CFS. Deepthi et al. (2016) also reported conidial and biomass inhibition of *F. proliferatum* after treatment with CFS from *Lactobacillus plantarum* MYS6. Likewise, *Lactobacillus plantarum* MYS44 suppressed the conidial germination and mycelial growth of *Aspergillus parasiticus* (Poornachandra Rao et al., 2019).

In the present study, *L. brevis* MYSN105 inhibited both mycelial biomass and conidial germination. This antifungal activity of LAB is a complex phenomenon mainly due to the high capacity of these bacteria to produce multiple antagonistic compounds that often act in synergy (Laitila et al., 2004). The CFS recovered from *L. brevis* MYSN105 had a potent inhibitory effect on *F. verticillioides* in maize seeds and *in vitro* cocultures, per *in vitro* reports on *Lactobacillus* species having antifungal activities (Muñoz et al., 2010; Voulgari et al., 2010; Gerbaldo et al., 2012). Finally, the strain *L. brevis* MYSN105 isolated from traditional fermented food Pozha showed antagonistic activity against *F. verticillioides*, suggesting a promising biocontrol potential.

## Conclusion

The present study identifies *L. brevis* MYSN105 isolated from fermented food Pozha as an effective biocontrol of the mycotoxigenic *F. verticillioides* that are natural contaminants of raw materials used in the food and feed production. Future studies with *L. brevis* MYSN105 are required to test their ability to inhibit fumonisin biosynthesis and test their application in biocontrolling other fungal contaminants and extending the shelf life of food and feed.

## Data Availability Statement

The datasets presented in this study can be found in online repositories. The names of the repository/repositories and accession number(s) can be found in the article/supplementary material.

## Author Contributions

MYS, RH, and RS designed the research work. RS, AG, and UJ performed the research activities. RS and WM analyzed the data and wrote the manuscript. WM, RH, and MYS edited the manuscript submitted. All authors have given their approval for publication and have no conflict of interest.

## Conflict of Interest

The authors declare that the research was conducted in the absence of any commercial or financial relationships that could be construed as a potential conflict of interest.
